# Association between Consumption of Fluoroquinolones and Carbapenems and Their Resistance Rates in *Pseudomonas aeruginosa* in Argentina

**DOI:** 10.1155/2022/3924212

**Published:** 2022-02-02

**Authors:** Silvia Boni, Gustavo H. Marin, Laura Campaña, Lupe Marin, Soledad Risso-Patrón, Gina Marin, Fernanda Gabriel, Alejandra Corso, Valeria Garay, Manuel Limeres

**Affiliations:** ^1^National Administration of Drugs, Food and Technology of Argentina, Avenida de Mayo 869, Ciudad Autónoma de Buenos Aires, Argentina; ^2^National Scientific and Technical Research Council, CCT La Plata. Calle 8 Nº 1467, La Plata, Buenos Aires, Argentina; ^3^National University of La Plata, Facultyof Medical Science, Calle 60 y 120, La Plata, Buenos Aires, Argentina; ^4^Scientific Research Commission, Calle 526 y Con. Gral Belgrano, La Plata, Buenos Aires, Argentina; ^5^National Ministry of Health, Av. 9 de Julio 1925, Ciudad Autónoma de Buenos Aires, Argentina

## Abstract

**Background:**

Irrational use of antimicrobials (ATMs) triggers microbial resistance (AMR) which has severe consequences for human health. ATM consumption varies among countries and within each territory. These data should be known, in order to perform local policies towards AMR reduction. This work aimed to expose the association of the level of consumption of carbapenems and fluoroquinolones with their resistance rates in *Pseudomonas aeruginosa* in Argentina.

**Method:**

Consumption of antimicrobials was expressed by defined daily dose (DDD)/1000 inhabitants for each ATM during one year period, discriminating by each country region. Resistance of *P. aeruginosa* to carbapenems/fluoroquinolones groups was recorded. Consumption/resistance ratio “R” was calculated for each region of the country, comparing results with other countries.

**Results:**

*P*. *aeruginosa* resistance rate to fluoroquinolone (F) was 26.4% in blood samples and 29.7% in urine samples, whereas resistance rates to carbapenems (C) were 19.9 and 17.7% in blood and urine, respectively. Correlation between consumption and resistance was demonstrated for both antimicrobials (C : *R* = 0.58; *p*=0.003 and F : *R* = 0.77; *p*=0.0001). Great fluctuations of resistance levels were seen among regions within the country, always correlating resistance with areas in which a higher level of ATM consumption was detected.

**Conclusion:**

*P. aeruginosa* resistance to fluoroquinolone/carbapenems in Argentina directly correlated with antimicrobial consumption levels. A great heterogeneity in resistance profile was observed among areas where ATMs were widely used. Global data at the national level might mask local realities that require specific health policies in order to control the irrational use of ATMs.

## 1. Introduction

Over the years, as science developed new antimicrobials (ATMs), microorganisms have developed mechanisms of defense against them. In this “speed race,” when we take a look at the small amount of new antibiotics developed in the last decades and the sophisticated antimicrobial resistance (AMR) mechanisms that bacteria had performed, it is clear who is winning the contest [1].

The AMR complexity forces countries to adopt policies that limit the use of certain ATMs, that the World Health Organization classified as “Watch” or “Reserve” groups, in an attempt to reduce the clinical and economic consequences for the health system. Factors such as the misuse and overuse of antimicrobials have accelerated the emergency and spread AMR [2]. AMR is associated to empirical treatment failure, aggravates morbidity, increases mortality, and has negative impact on the costs of health care [3]. AMR developed by microorganisms, expresses their ability to grow in the presence of certain ATM groups designed to inhibit them.

*Pseudomonas aeruginosa*, for example, is a Gram-negative aerobic bacterium, motile, non-spore-forming rods, oxidase-positive and lactose nonfermenters [4], that has a great capacity to develop resistance. It could be said that *P.aeruginosa* represents one of the most concerning pathogens involved in antibiotic resistance [5], being a highly adaptable opportunistic pathogen which is ubiquitously present in the environment. This bacterium is naturally resistant to many antimicrobials and can acquire antibiotic resistance through mutations in chromosomal genes and lateral gene transfer [6]. It could be associated with different types of human infections and because of its emerging multidrug-resistant strains, these types of infections are considered major global problem for the public health [7].

The selection of the most appropriate antibacterial treatment against *P. aeruginosa* becomes then an essential decision in order to optimize clinical results and reducing patient's morbidity and mortality as well as shortening the length of hospital stay and the spread of the pathogen in the hospital. However, this choice is not easy, given the ability of these bacteria to display all their resistance mechanisms to inactivate a variety of antimicrobials, even after starting antibiotic treatment [8].

Since *P. aeruginosa* is intrinsically resistant to many antimicrobial drugs, newly therapeutic groups such as carbapenems and fluoroquinolones became crucial in clinical management. Unfortunately, these antimicrobials are no exempt of resistance.

Carbapenem is the beta-lactams antibiotic with the greatest spectrum of action, and in many cases, it is considered the last therapeutic option against *P. aeruginosa* infections. However, acquired resistance to carbapenems exists and results from the presence of different mechanisms, including enzymatic inactivation and decreased antimicrobial concentration at the target site [9]. The relative importance of each resistance mechanism is variable and, frequently, more than one mechanism coexists [10]. Data from Europe show that the prevalence of resistance of *Pseudomonas* to carbapenems is between 17 and 22% in both blood and urine samples [11, 12].

Fluoroquinolones are another antimicrobial group classified by the WHO as “Watch” with a broad spectrum but widely prescribed to treat infections caused by *Pseudomonas* [13]. Bacterial resistance for fluoroquinolones has been demonstrated in various clinical trials [14]. The prevalence of *P. aeruginosa* resistance in the USA increased from 15% to 41% in the last ten years [13].

There exists well-known evidence that increased resistance to both carbapenems and fluoroquinolones is linked to the excessive use of these antibiotics [15].

In this sense, the World Health Assembly, in its global action plan to combat AMR [7], recognized that an important tool to fight against resistance has been the development of national strategies for the rational ATM consumption (avoiding self-medication and dispensing without prescription and control the overuse), the appropriate use (indication according to diagnosis), the adequate use (correct selection of administrations routes, doses, and duration), and reduction of unnecessary costs associated with irrational use [15].

Countries with high antimicrobial consumption typically have high levels of AMR [16, 17]. Indeed, the increased detection and spread of carbapenemase-producing bacteria in Latin America and the Caribbean in recent years illustrates the seriousness of the AMR problem and the imperative need to be aware and engage governments to adopt local policies to regulate ATM consumption [18].

This study aimed to describe and analyze antimicrobial consumption of fluoroquinolones and carbapenem and its association with *P. aeruginosa* resistance in Argentina, in order to perform public policy actions focusing on those regions with the highest level of consumption/resistance problems.

## 2. Materials and Methods

### 2.1. Study Design

We conducted a descriptive, retrospective drug utilization research study with an analytical phase in order to determinate the drug consumption of fluoroquinolone and carbapenem groups and its relation to *P. aeruginosa* resistance patterns in Argentina. The study period went from January 1 to December 31, 2018.

We compiled and analyzed data of consumption from all sales of ATM products performed by the pharmaceutical industry in 23 Argentine provinces and the Autonomous City of Buenos Aires (CABA) between January and December 2018. The study included all the sales made in Argentina limited to human medicine applications, which is the entire universe of fluroquinolones and carbapenems from the “Watch” group commercialized in the country according to their ATC and “Aware” classifications [19].

We collected information about the type of ATM according to the anatomical, therapeutic, chemical (ATC) classification system; consumption of defined daily doses (DDDs) as defined by the World Health Organization (technical unit of measurement of drug consumption known as a defined daily dose [19], which expresses the daily dose of a drug for its main indication in adults); geographical territory in which consumption/sale distribution was registered; and data related to the level of resistance for the different ATMs (global and by each region), according to the national registry generated periodically by the Malbran Institute [20].

The following indicators were considered for the analysis [21]:

Global consumption of antimicrobials in Argentina was expressed in DDD per 1,000 inhabitants per day (DID). The calculation was made for each drug during the period analyzed, according to the formula of DID provided by the WHO:

DID was calculated by computerized methods using a WHO/GLASS program designed for this purpose.
Population exposed (PE) to antimicrobials was calculated for each active principle during the period analyzed, according to the formula: 
  (2) $$\begin{array}{c} \frac{\text{PE}=\text{DID}\times \text{no}.\text{ of inhabitants}}{1000}\text{.} \end{array}$$
(i) DDD: technical unit of measurement of drug consumption known as defined daily dose, which expresses the daily dose of a drug for its main indication in adults.
(ii) DID: it expresses the number of inhabitants of every 1000 that consume a DDD every day of a certain drug.
(iii) Consumption: quantity of medicines, in any of their forms of presentation, sold by the provincial warehouses to the health units for their use.
(iv) Population exposed (PE): number of patients exposed to a certain medicine. It is calculated from the DID.

The information on ATM resistance of priority pathogens was expressed in percentage. *P. aeruginosa* were collected and isolated from urine and blood samples. The data were analyzed and provided by the Malbran Institute which is the national reference laboratory for bacterial resistance, and it is the main institution for the performance of this type of diagnosis in Argentina. It also represents the international reference laboratory for the AMR network of the World Health Organization (WHONET).

Bacterial sensibility to carbapenems and fluoroquinolones was evaluated by the disk diffusion method (Baeur–Kirby) following the Clinical and Laboratory Standards Institute (CLSI) recommendations [22]. For this work, the analysis was limited to *P. aeruginosa* pathogen because it is one of the bacteria with the highest prevalence of severe infections among inpatients in Argentina.

The data were analyzed with the WHONET5.6 software. Results were expressed as % nonsensitivity (NS) (% I + % R). The data obtained were grouped and stratified by federal states and regions due to their different historical, geographical, social, economic, and sanitary situations.

After recording resistance values observed during 2018, the data were compared with levels of antimicrobial consumption detected either globally or in each region of the country.

Additionally, in order to compare consumption/resistance ratios obtained in Argentinian regions with other territories, the published data from European countries were incorporated into the final analysis.

## 3. Statistical Analysis

As mentioned before, consumption was expressed in DDD/DID and resistance was expressed in percentage of pathogen resistance to each antimicrobial group.

The Kolmogorov–Smirnov test (*p* value >0.05) and Bartlett's test (*p* value <0.05) were executed to determine the normal distribution and homogeneous variances of the data. The correlation and linear regression analysis were performed. Linear regression analysis was used to evaluate the trends of antibiotic consumption and antibiotic resistance. A *p* value of <0.05 was considered as statistically significant.

For data analysis, we used the open access software “R” (version 4.0.0), with its default packages, tidyverse, and agricolae, for statistical computing and graphics and ggplot package for the development of the graphs.

## 4. Results

Global antimicrobial consumption data of Argentina were obtained from information about all sales performed by the pharmaceutical industry that commercialized their products (produced and imported) for human use in the national territory. Among the carbapenem group, the average overall consumption was 0.021 DDD/1000 inhabitants, where meropenem and imipenem were the most consumed drugs among the group (Table 1).

In regard to the fluoroquinolone group, ciprofloxacin was the most consumed drug, followed by levofloxacin (Table 2).

Concerning *P. aeruginosa* resistance, the data from either blood and urine samples were collected by the WHONET surveillance program during the study period. It was demonstrated that, in average, global resistance levels to fluoroquinolone were 26.42% (SD = 6.8) and 29.72% (SD = 5.5), while regarding to carbapenem were 19.95% (SD = 9.1) and 17.73% (SD = 5.6) in blood and urine samples, respectively.

It is noteworthy that the data showed a great heterogeneity among geographical regions (Table 3). The analysis of resistance to fluoroquinolones revealed statistically significant differences between the capital region (CABA) compared with other regions (*p* value<0.01) and to carbapenem between CABA and regions of North and Cuyo (*p* value< 0.01).

The resistance level of *P. aeruginosa* showed great differences among the regions. When compared data from Argentina with most European countries [19], it could be said that levels of resistance were considered “high” in all regions of Argentina, either for carbapenem (average level of resistance was 16.8% SE ± 10.05) or for fluoroquinolone (average level 20.1% SE ± 10.43), which were similar to data shown by Spain, Czech Republic, Greece, or Romania.

**Figure 1:**
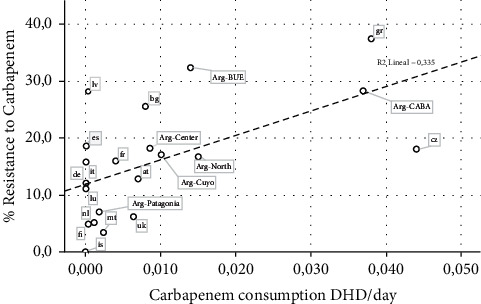
Carbapenem consumption and *Pseudomonas aeruginosa* resistance data (according to Argentinian region compared with European countries). Countries are named with their official country codes [23].

It was observed a significant correlation (*p*=0.003) between carbapenem consumption levels and the bacterial resistance to this antimicrobial (R = 0.58), showing a determination coefficient of 0.34 (SE = 8.41) (Figure 1). In the capital region of Argentina (CABA), high consumption and high resistance levels were detected, only similar to data reported by Greece or Czech Republic (20–23), while in other regions such as Patagonia, the consumption/resistance ratio profile was more like scenarios seen in Finland or the Netherlands (Figure 1).

**Figure 2:**
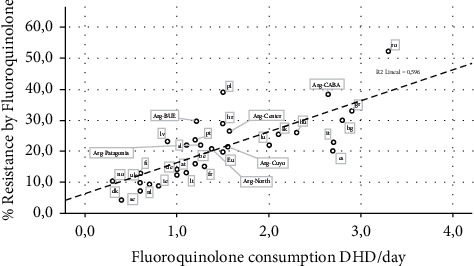
Fluoroquinolone consumption and *Pseudomonas aeruginosa* resistance data (according to Argentinian region compared with European countries). Countries are named with their official country codes [23].

Concerning the fluoroquinolone group, the consumption/resistance correlation was also significant (*p* < 0.0001) with an *R* value of 0.77 and determination coefficient of 0.60 (SE = 6.74) (Figure 2).

Again, high in CABA (similar to the ratio found in countries such as Bulgaria or Greece) (Figure 2), while in other regions, this ratio was low as the great majority of most developed countries.

## 5. Discussion

Antimicrobial resistance is a complex public health challenge impacting all regions of the world. In addition to being a natural evolutionary phenomenon, AMR is increasingly being accelerated by selective pressure exerted as a result of the use and misuse of antimicrobials [23, 24]. Knowing the reality about the main bacteria that develop resistance, the mechanisms that generate that AMR and the level of consumption of antimicrobials that induce resistance become essential data for health authorities [25–28].

Among all bacteria, *Pseudomonas aeruginosa* are considered by the WHO as one of the 5 main threats to the public health [29]. The explanation for their categorization is that *P. aeruginosa* are widespread in the environment and commonly occurring in soil and water. They are capable of growth in low-nutrient situations and can grow in water in distribution systems if they gain access and on materials used in domestic plumbing situations. This is why *P. aeruginosa* may colonize taps and grow on surfaces, such as plastic pipes, in drink vending machines. In the last years, the emergence of multidrug-resistant (MDR) *P. aeruginosa* located these bacteria in the top of the list, becoming a major public health issue worldwide [29].

*Pseudomonas aeruginosa* are also considered one of the leading causes of severe infections associated with high mortality rates and with high difficulty to treat, as the repertoire of useful antipseudomonal agents is limited since they have capacity to develop resistance to all known antibiotics [30]. Although the *Pseudomonas aeruginosa* infection is well-known and mostly found in hospitals and nursing care facilities, many cases are also reported outside these boundaries [31].

However, the information available about *P. aeruginosa* resistance to the main antimicrobials groups such as carbapenems or fluoroquinolones is limited to health institutions (hospitals) while data from global countries are still meagre. Data extracted from the hospital level demonstrated a correlation between carbapenem use and carbapenem resistance rates of *P. aeruginosa* [32]. In this bacterium, the range of antibiotics used against resistance was demonstrated by a positive correlation between resistance and consumed antibiotics (coefficient 1.77; 95% confidence interval, 0.58 to 2.96; *p* < 0.01) [33]. However, a great disparity was seen among hospitals enrolled in the studies [33]. Concerning fluroquinolone consumption and resistance rate at the hospital level, a large study in Chinese institutions demonstrated that *Pseudomonas aeruginosa* resistance is correlated with consumption of these antibiotics (*r* = 0.260, *p* < 0.01) and FQs (*r* = 0.319, *p* < 0.01) [34]. Again, disparities were demonstrated among hospitals that participated in the study [34]. The type of *P. aeruginosa* resistance to fluoroquinolones is also heterogeneous: mutations in the genes encoding bacterial DNA topoisomerase II and topoisomerase IV, overexpression of active efflux systems, and plasmid carrying quinolone-resistance genes are only three of the several ways of AMR developed by *P. aeruginosa* [35, 36].

Policies and strategies to control AMR and antimicrobial misuse are wide different according to the level of where they are used [37]. Antibiotic stewardship in hospitals is a key strategy for controlling antibiotic resistance. Guidelines, recommending formularies that restrict prescriptions, and special medical and pharmacist training become core strategies to solve local antibiotic use and resistance problems. However, other tools should be considered when global national consumption is involved. Diversification in antibiotic use, control of antimicrobials prescriptions in certain regions, or proposed focalized health professional trainings may can help avoid the selection pressure that might result from the use of a list of antibiotics in a country. Hence, information of consumption in global countries as the one provided from this research becomes essential.

Antimicrobial consumption has been usually well-controlled in certain high-income countries, whereas it has been disproportionally high in low- and middle-income countries [26]. In Argentina, this statement was confirmed for the beta-lactams group, but data about other antimicrobial groups are still missing [38]. Information was an important tool to modify irrational antimicrobial consumption in many countries [39–41]; however, in Latin America, these data are still lacking [26]. Although, there is no doubt that a high level of antimicrobial consumption is directly related to the level of bacterial resistance [42], antimicrobial usage patterns data are required to understand local resistance levels [43, 44], including its use in animal production [45, 46].

The results obtained in the present study about fluoroquinolone and carbapenem groups showed that overall consumption of these antimicrobials in Argentina is considered mild-high level, with a big heterogeneity among each region. This information is comparable with data reported by other authors, that demonstrated that in the last decade, existed an increase of all antibiotics consumption in low- and middle-income countries, four times more that in high-income countries (56 vs 15%) [47, 48]. Specifically for fluoroquinolones, this increase was 12% of total DDDs, reaching the consumption of high-income countries (which reduced this consumption by 1.2% during the same period) [48]. On the other hand, although carbapenem consumption rate duplicated in the last decade in low middle-income countries, its consumption levels still remain far below the consumption rates in high-income countries (0.03 DID vs 0.1 DID, respectively) [47, 48].

Regarding *P. aeruginosa* resistance, our work showed wide differences among areas of the country in which the study was carried out. It should be noticed that in most urban areas, antimicrobial resistance was always high. The data from Table 3 demonstrated how, in most populated regions such as CABA or Buenos Aires, the level of resistance either to fluoroquinolone or carbapenem was much higher than that in regions such as Patagonia or North, where the density of inhabitants is very low. This pattern was observed for *P. aeruginosa* isolated from both urine and blood samples.

The analysis of carbapenem and fluoroquinolone consumption data together with the levels of resistance of *P. aeruginosa* to these antimicrobials reflected the degree of the association between both variables. Results obtained aware about the ominous consequences of the high consumption of antimicrobials that should be used with caution, like all those belonging to the “Watch” group.

Observations about consumption/resistance correlation ratio detected in our study were similar to those previously reported at the hospital level by Yang et al. [34]. According to these authors, in China, among 145 hospitals, the median resistance rate of *P. aeruginosa* to fluoroquinolones was 22.4% (7.7–65.2%), data similar to results obtained in our research (26.4% (20.7–38.3)). When this resistance was correlated with the overall country consumption of fluoroquinolones, results were similar to the ones described in China (*r* = 3.11 *p* < 0.01 vs *r* = 0.319, *p* < 0.01); however, in certain regions such as CABA, the relation was clearly increased (*r* = 5.744 *p* < 0.001 vs *r* = 0.319 *p* < 0.01).

Same aspect was seen regarding consumption of carbapenems and *P. aeruginosa* resistance level to this group of antimicrobials in studies reported by Plüss-Suard et al. [32]. From these research studies, we know that hospital resistance to carbapenems was correlated to the level of consumption in both reports (*r* = 0.305, *p* 0.045 and *r* = 0.463, *p* < 0.01, respectively), data that match with results obtained in our study (*r* = 0.485, *p* < 0.01).

Data extracted from analysis of different areas within Argentina demonstrated that although the country shows a moderate/high consumption/resistance ratio profile, certainly, regions such as the capital area (CABA) had much higher levels of consumption and also show an increase in *P.aeruginosa* resistance levels either to carbapenems or to fluoroquinolones. In very sparsely populated regions, such as Northern states or Patagonia, the consumption of the “Watch” antimicrobial group such as fluoroquinolone or carbapenem is better controlled, which may explain the low resistance level observed there. This situation might change in the next years since although policies of rational use of medicines in health institutions are currently adopted, recently, there exists an indiscriminate use of antimicrobials for animal husbandry, an aspect that may change the profile of these regions in the future. Results of high “Watch” antibiotic consumption observed in most populated urban areas of Argentina can be compared with the escalation detected in low-income and middle-income countries in the last years, which exhibited an increase of 165·0% (from 2·0 to 5·3 DIDs) in relation with high-income countries (27·9%; from 6·1 to 7·8 DIDs). [48].

When the global data of Argentina are compared with other countries such as the ones of the European Union [49], it could be said that its levels of consumption/resistance are similar to those countries with a much higher ratio; however, it remains a great disparity among Argentinian regions in relation to *P. aeruginosa* resistance and “Watch” antimicrobials consumption, reflecting the existence of several realities within the same national territory (Figures 1 and 2). This situation observed in Argentina should make us reflex about the fact that these same realities might be existing in other countries and that mean global data could be hiding local realities that need focal decisions towards the control of the irrational use of antimicrobials.

## 6. Conclusions

The analysis performed about the global use of “Watch” antimicrobial group such as fluoroquinolones/carbapenems and *P. aeruginosa* resistance level to these drugs demonstrated that Argentina has an over all moderate/high consumption/resistance ratio with a heterogeneous pattern among regions within the national territory. These different realities should be taken into account at the time to raise local health policies for AMR control.

## Figures and Tables

**Table 1:** Level of global carbapenem consumption according to the drug type during 2018.

Type of carbapenem	Consumption level
Ertapenem	0.0067
Imipenem	0.024
Meropenem	0.033

Consumption during 2018 was measured as defined daily dose by each 1000 inhabitant.

**Table 2:** Level of global fluoroquinolone consumption according to the drug type during 2018.

Type of fluoroquinolone	Consumption level
Ciprofloxacin	0.7838
Levofloxacin	0.51
Moxifloxacin	0.0065
Norfloxacin	0.1410

Consumption during 2018 was measured as defined daily dose by each 1000 inhabitant.

**Table 3:** The carbapenem and fluoroquinolone resistance rates in *Pseudomonas aeruginosa* according to each geographical region of Argentina during 2018.

Type of resistance	S	Capital city (CABA)			Buenos Aires (BUE)			Center (CEN)			Cuyo region (CUYO)			North (NOR)			Patagonia (PAT)		
		I	NRI	%	I	NRI	%	I	NRI	%	I	NRI	%	I	%	NRI	I	NRI	%
Resistance to fluoroquinolone	U	340	124	36.5	288	95	33.0	190	57	30.0	123	29	23.6	162	37	22.8	131	42	32.4
	B	154	59	38.3	145	43	29.7	53	14	26.4	42	9	21.4	87	18	20.7	41	9	22.0
Resistance to carbapenem	U	336	83	24.7	292	50	17.1	192	33	17.2	120	15	12.5	162	18	11.1	130	31	23.8
	B	159	45	28.3	145	47	32.4	55	10	18.2	41	7	17.1	90	15	16.7	43	3	7.01

S: sample type; U: urine sample; B: blood sample; I: isolations; NRI: no. resistant isolations; CABA: capital city of Buenos Aires (capital of Argentina); BUE: Buenos Aires (Buenos Aires Province); CEN: center (Cordoba, Santa Fe, Entre Rios); CUYO: Cuyo region (Mendoza, San Juan, San Luis); NOR: North (Jujuy, Salta, Tucuman, Catamarca; La Rioja, Formosa, Chaco, Santiago del Estero; Corrientes *y* Misiones).

## Data Availability

The data from original ANMAT, used to support the findings of this study, were supplied by ANMAT-Ministry of Health, so it cannot be made freely available. Database is available on request through a contact of the corresponding author of this paper. Requests for access to these data should be made by e-mail to Dr. Gustavo Marin gustavo.marin@anmat.gob.ar.
